# Ophthalmologic compromise following SARS-CoV-2 vaccinations

**DOI:** 10.1186/s12348-022-00318-x

**Published:** 2022-11-24

**Authors:** Josef Finsterer

**Affiliations:** Neurology & Neurophysiology Center, Postfach 20, 1180 Vienna, Austria

**Keywords:** SARS-CoV-2, Vaccination, Ophthalmology, Vision, COVID-19

Letter to the Editor

We read with interest the article by Murgova et al. about five patients with ophthalmologic complications after a SARS-CoV-2 vaccination [1]. Patient-1 experienced right herpetic keratitis and iridocyclitis, patient-2 left anterior uveitis, patient-3 retinal detachment, retinal necrosis, and vitritis, patient-4 anterior ischemic optic neuropathy (AION), and patient-5 left ptosis after subarachnoid bleeding (SAB) (Table 1) [1]. The study is promising but raises concerns that should be discussed.

**Table 1:** Clinical data of the five patients presented in the report

Patient	1	2	3	4	5
Age (y)	56	89	50	45	53
Sex	male	female	male	male	female
Vaccine brand	1. AZV	2. BPV	2. BPV	2. BPV	BPV
LVOD (d)	7	21	8	10	14
POD	herpetic keratitis, Iridocyclitis	right central vein occlusion, cataract, glaucoma	herpes uveitis iridocyclitis, COVID-19	non-arteritic AION	none
BCVA	0.02/1.0	hm/0.09	1.0/hm	1.0/1.0	1.0/1.0
Diagnosis	exacerbation of herpetic keratitis, iridocyclitis	anterior uveitis	retinal necrosis detachment vitritis	AION	ptosis after SAB ophthalmoparesis
Side	right	bilateral	left	left	left
Therapy	AV, ST	ST, antiglaucoma therapy	AV, ST, surgery	vasodilators antiplatelet agents	nm
Outcome (BCVA)	0.7/1.0 (1 m)	0.4/0.4 (4 m)	nm/nm	1.0/1.0	nm/nm

*AION* Anterior, ischemic optic neuropath, *AV* Acyclovir, *AZV* AstraZeneca vaccine, *BCVA* Best corrected visual acuity, *hm* Hand movement, *LVOD* Latency between vaccination and ophthalmologic disease, *nm* Not mentioned,: previous ophthalmologic disease, *SAB* Subarachnoid bleeding, *ST* Steroids

A causal relationship between the vaccination and the ophthalmologic abnormality remains unproven in patient-1. Herpetic keratitis and iridocyclitis in a patient with a history of keratitis and iridocyclitis can also have many other causes and may not necessarily be triggered by the vaccination. A causal relationship between vaccination and SAB in patient-5 has also not been proven. Arterial hypertension that may have caused rupture of a pre-existing aneurysm may be due to several other causes. We should know whether patient-5 already had arterial hypertension before SAB and whether there were other reasons for arterial hypertension. In patient-3, a recurrence of the herpetic infection could also be responsible for the acute deterioration.

Ptosis, ophthalmoparesis, and pupillary dilatation after SAB is not uncommon and rather a neurological than an ophthalmologic complication after SAB. Unfortunately, nothing is reported about the outcome of these deficits and of SAB [1].

Acute infection with SARS-CoV-2 was not ruled out in all five patients. Since we are living in the time of an undulating pandemic and the ophthalmological abnormalities described in the five index patients can also be the result of an acute SARS-CoV-2 infection, such an infection must be ruled out by documenting a negative PCR test.

The spectrum of ophthalmologic side effects after SARS-CoV-2 vaccinations is broader than mentioned in the introduction and not only includes uveitis, maculopathy, neuro-retinopathy, or Vogt-Koyanagi-Harada syndrome [1]. Ophthalmologic complications after SARS-CoV-2 vaccinations additionally reported include neuromyelitis optica (NMO) [2], myelin associated glycoprotein (MOG) associated disease [3], acute disseminated encephalomyelitis (ADEM) with optic neuritis [4], non-specific optic neuritis, and exacerbation of multiple sclerosis (MS) [5].

Although a causal relationship between vaccination and the reported ophthalmologic abnormalities remains unproven, it is supported and suggested by the plausibility of biological mechanisms analogous to those reported after other types of vaccination. Furthermore, consistency and reproducibility were demonstrated by other studies reporting similar observations, which also showed that some of the patients who developed an ocular inflammatory event after the 1st dose, developed a similar event after the 2nd dose [6].

Overall, the interesting study has limitations that call the results and their interpretation into question. Clarifying these weaknesses would strengthen the conclusions and could improve the study. Before blaming SARS-CoV2- vaccinations for acute ophthalmologic disease, all differentials need to be appropriately ruled out.

## Data Availability

Not applicable.
